# Self-Reported Changes in Oral Hygiene Habits among Adolescents Receiving Orthodontic Treatment

**DOI:** 10.3390/dj7040096

**Published:** 2019-10-01

**Authors:** Sandra Petrauskiene, Natalia Wanczewska, Egle Slabsinskiene, Gintare Zemgulyte

**Affiliations:** 1 Clinic for Preventive and Paediatric Dentistry, Lithuanian University of Health Sciences, Luksos-Daumanto 6, 50106 Kaunas, Lithuania; nataliawanczewska@gmail.com (N.W.); egleslabsinskiene@yahoo.com (E.S.); 2 Neurology Department, Academy of Medicine, Lithuanian University of Health Sciences, Eiveniu 2, 50103 Kaunas, Lithuania; gintare.zemgulyte@lsmu.lt

**Keywords:** oral hygiene habits, orthodontic treatment, adolescents

## Abstract

The prevalence of malocclusion and a need for orthodontic treatment is high. Orthodontic appliances increase biofilm accumulation by expanding plaque retention sites. The aim of this study was to investigate the self-reported changes in oral hygiene habits among adolescents receiving orthodontic treatment. A cross-sectional study of 291 patients aged 10–17 years (mean (M) = 12.98; standard deviation (SD) = 2.36) was conducted in the Department of Orthodontics, Lithuanian University of Medical Sciences (LSMU) Hospital (Kaunas, Lithuania) during the fall semester (October–January) of the 2017/2018 study year. An anonymous self-administered questionnaire covered background information, experience of orthodontic treatment, oral hygiene habits and the seeking of professional dental care. Statistical data analysis was performed using SPSS version 22. To establish relationships between categorical variables, Chi-squared tests (χ^2^) were used. A *p*-value ≤ 0.05 was set to indicate statistically significant difference. The univariate logistic regression analysis evaluated the probability of an event given a certain risk indicator, including odds ratio (OR) and its confidence interval (95% CI). Associations were found between the usage of auxiliary measures (OR = 1.797 (1.118–2.887), *p* = 0.015), tongue cleaning (OR = 1.712 (1.059–2.767), *p* = 0.028), mouth rinsing after meals (OR = 1.707 (1.048–2.781), *p* = 0.032) and experience of orthodontic treatment, respectively. More orthodontic patients underwent professional oral hygiene regularly than non-orthodontic patients (*p* = 0.024). More patients with fixed orthodontic appliances reported significantly changed oral hygiene habits, while more orthodontic patients with removable appliances did not change their oral hygiene habits.

## 1. Introduction

Currently, patient demands and expectations regarding aesthetics are increasing remarkably [1]. Although malocclusion is not a disease, this morphological variation is often defined as a deviation from beauty norms, which can cause low self-esteem and insufficient self-confidence in people due to frequent teasing experiences and dissatisfaction [2,3,4]. Accomplished orthodontic treatment can improve multidimensional attitudes toward body image and mental health status [5]. Subsequently, an inappropriate aesthetic is the main reason for patients to undergo orthodontic treatment, while medical and dental indications are less prevalent as a reason for orthodontic treatment [6,7,8].

The factors of proper oral hygiene, correct diet and regular dental check-ups are key to prevent not only hard dental tissue demineralization, but also periodontal diseases during orthodontic treatment [9,10]. Orthodontic appliances deteriorate the self-cleaning of teeth provided by the tongue, cheek and lip muscles during mastication, and they increase biofilm accumulation by expanding plaque retention sites around the components of fixed appliances attached to the teeth [11,12,13,14,15].

Considering different methods of orthodontic treatment, fixed and removable orthodontic appliances are used for patients. Furthermore, self-cleaning oral hygiene measures and tooth brushing techniques vary according to the type of orthodontic treatment received. Several authors agree that patients receiving orthodontic treatment with removable appliances can maintain good oral hygiene more easily than with fixed appliances, because these appliances can be taken out. Consequently, the patient’s teeth and the removable orthodontic appliance can be brushed, under ideal conditions [16,17,18]. Meanwhile, patients with fixed orthodontic appliances face a special challenge in removing plaque mechanically because of trapped food around the brackets and other fixed components [19]. In addition, there are conflicting results surrounding the supposedly better facilitation of oral hygiene when patients receive orthodontic treatment with self-ligated brackets and clear aligners. Chhibber et al. [20] reported that the type of brackets, such as self-ligated, elastomeric-ligated or clear aligners (Invisalign), is not an essential factor affecting the status of oral hygiene. Moreover, orthodontic treatment with fixed appliances usually lasts an average of 24 months, and continuous substandard oral hygiene habits may lead to hard tissue demineralization [13,21]. Orthodontic treatment is usually performed on teenagers. Adolescent patients often have suboptimal manual ability, low motivation and poor long-term compliance regarding good oral hygiene maintenance, and so therefore oral diseases of the hard and soft tissues develop for patients receiving orthodontic treatment [22,23,24].

Patients presenting good attendance, following the orthodontist’s recommendations, caring for appliances and maintaining proper oral hygiene are judged as “adherent” and show more favorable clinical orthodontic outcomes than “non-adherent patients” [25,26].

The aim of this study was to assess attitudes toward oral hygiene and the self-reported changes in oral hygiene habits among patients receiving orthodontic treatment with removable and fixed appliances.

## 2. Materials and Methods

A cross-sectional study of assessment attitudes toward oral hygiene and self-reported changes in oral hygiene among adolescents receiving orthodontic treatment was carried out in the Department of Orthodontics, Lithuanian University of Medical Sciences (LSMU) Hospital (Kaunas, Lithuania) during the autumn semester (October–January) of the 2017/2018 study year. The study was approved by the Bioethics Center of the Lithuanian University of Health Sciences (No. BEC-OF-14) on the 3 October 2017. We started to collect the data after this approval. 

The principle investigator (NW) asked all adolescents (N = 300) attending the Department of Orthodontics to complete an anonymous self-administered questionnaire during the dental appointment. The aim of the study was explained to parents and participants before filling in the questionnaire. Participation was voluntary and anonymous; thus, the return of a completed questionnaire and the consent signed by parents was considered acceptance to participate. Overall, 291 completed questionnaires were returned.

The sample size was calculated using Paniott’s formula with an error of 0.05% based on the number of patients (10–17 years old) attending the Department of Orthodontics [27]. By using this formula, it was determined that no less than 300 10–17-year-old adolescents needed to be included in the study.

### 2.1. Subjects

Subjects were patients aged 10–17 years old attending the Department of Orthodontics at LSMU Hospital. The inclusion criteria of subjects were patients aged 10–17 years old attending the Department of Orthodontics at LSMU Hospital and willingness to participate, while the exclusion criteria was patients aged <10-years-old and ≥18-years-old attending the Department of Orthodontics at LSMU Hospital.

Participants were dichotomized, according to age, into two groups of 10–12 and 13–17 years due to several reasons such as dentition status and peculiarities of child behavior and development. Considering the dentition status, children aged 10–12 years old have mixed dentition and adolescents aged 13–17 will have permanent dentition. In addition, there are differences in child behavior management and development between these age groups (pre-teenagers and adolescents).

Considering orthodontic treatment, participants were grouped into the following groups: patients not receiving orthodontic treatment (control group) and patients receiving orthodontic treatment (study group). Then, the study group was divided into patients receiving orthodontic treatment with fixed appliances (braces, functional orthodontic appliances and retention appliances) and patients receiving orthodontic treatment with removable appliances.

A total of 291 participants were enrolled in this study: 170 in the study group (48 with fixed appliances (30 with braces, 12 with functional appliances and 6 with retention appliances) and 122 with removable appliances) and 121 in the control group.

The response rate was 97%.

### 2.2. The Questionnaire

A questionnaire was developed by authors (P.S. and W.N.). An anonymous Lithuanian self-administered questionnaire consisting of 26 items covered background information (gender, age, place of residence), experience of orthodontic treatment (not receiving orthodontic treatment, receiving orthodontic treatment such as braces, a removable appliance, a functional appliance or a retention appliance), oral hygiene habits and dietary habits, and the seeking of professional dental care.

The question about the frequency of tooth brushing presented three options: irregularly, once a day, and twice a day or more. The item about the duration of tooth brushing had the following options: brushing for up to 1 min, brushing for 2 min, brushing for at least 2 min. Later, these options were dichotomized into two groups: brushing for up to 1 min and brushing for at least 2 min.

Questions about oral hygiene measures enquired about the type of toothbrush used (manual or powered) and toothpaste (with fluoride, fluoride free, and do not know). Participants were asked if they cleaned their tongue (yes, no). The item about the usage of self-care adjunctive aids had four options of answers: floss, interdental brush, irrigator, and do not use. Subsequently, these options were dichotomized into two groups: yes (if they use at least one interproximal measure: floss, interdental brush or irrigator) and no (do not use). The question about mouth rinsing after meals had the following options: always, often, seldom and never. Later, these options were regrouped into yes (always, often and seldom) and no (never).

Items about professional dental care included questions about habitual dental attendance (once a year, twice a year and irregularly) and professional oral hygiene attendance (every 3 months, every 6 months, once a year and do not attend). Later, the options of professional oral hygiene were dichotomized into two groups: yes (every 3 months, every 6 months, and once a year) and no (do not attend).

Additionally, patients receiving orthodontic treatment were asked to assess their change in oral habits during orthodontic treatment (no changes, do not know, changed a bit, and changed a lot).

### 2.3. Statistical Analysis

Statistical data analysis was carried out using SPSS (Statistical Package for the Social Sciences for Windows, Chicago, IL, USA) version 22.

To establish relationships between categorical variables, Chi-squared tests (χ2) were used. The means of age and standard deviations (SD) were calculated. A *p*-value ≤ 0.05 was set to indicate statistically significant differences.

Univariate logistic regression analysis was used to evaluate the probability of an event (tongue brushing, usage of auxiliary measures and mouth rinsing after meals) given a certain risk indicator (patients receiving orthodontic treatment), including odds ratio (OR) and its confidence interval (95% CI).

## 3. Results

Overall, patients were almost equally distributed by age group (10–12-year-olds—50.2%, and 13–17-year-olds—49.8%). The mean age among participants was 12.98 (2.36) years (Table 1). Considering the place of residence, a majority of patients reported living in an urban area (72.9%, *p* = 0.285). Overall, more girls than boys (56.8% vs. 43.2%) participated in the study (*p* = 0.397, Table 1).

Overall, a majority of participants (61.6%) brushed their teeth at least twice a day. Although more patients receiving orthodontic treatment with fixed appliances (75.0%) brushed their teeth at least twice a day than the control group (57.0%), it did not differ statistically (*p* = 0.171, Table 2). In addition, 80.8% of all participants brushed their teeth for at least 2 min, and a higher prevalence was noticed in the control group (85.0%) than among patients receiving orthodontic treatment (*p* = 0.220), respectively (Table 2).

Almost all participants (95.5%) used manual toothbrushes (*p* = 0.899, Table 2). Considering the type of toothpaste, about half of patients (47.9%) did not know whether the toothpaste that they used contained fluoride (*p* = 0.926, Table 2).

Considering oral health self-care adjunctive aids, merely half (49.5%) of patients used at least one auxiliary oral hygiene measure. Subsequently, statistically significantly more participants living in rural areas (55.6%) did not use any auxiliary oral hygiene measure (*p* = 0.017). Furthermore, a higher share of patients receiving orthodontic treatment used auxiliary oral hygiene measures (56.0%) than in the control group (40.8%, *p* = 0.015, Table 3). Statistically significant associations were found between usage of auxiliary measures and experience of orthodontic treatment ((OR = 1.797 [1.118–2.887]), *p* = 0.015, Table 4).

Results showed that significantly more boys than girls (66.7% vs. 50.6%) did not clean their tongue (*p* = 0.006). In addition, significantly more participants receiving orthodontic treatment cleaned their tongue than patients not receiving orthodontic treatment (47.6% vs. 34.6%, *p* = 0.028, Table 3). Statistically significant associations were found between tongue cleaning and experience of orthodontic treatment (OR = 1.712 (1.059–2.767), *p* = 0.028, Table 4).

Overall, 62.1% of participants had a habit of rinsing their mouth after meals. Subsequently, this habit was more prevalent among patients receiving orthodontic treatment with fixed appliances (79.5%, *p* = 0.013, Table 2). Patients receiving orthodontic treatment had a 1.7 times higher probability of having a habit of rinsing their mouth after meals than control group patients (Table 4).

This study showed that more boys than girls had regular dental appointments once a year (54.1% vs. 53.5%, *p* = 0.05). In contrast, statistically significantly more participants living in rural areas reported irregular dental appointments compared to patients living in urban areas (21.7% vs. 15.9%, *p* = 0.035). Consequently, significantly more patients receiving orthodontic treatment (45.5%) had professional oral hygiene appointments than patients in the control group (31.5%, *p* = 0.024, Table 3).

**Figure 1:**
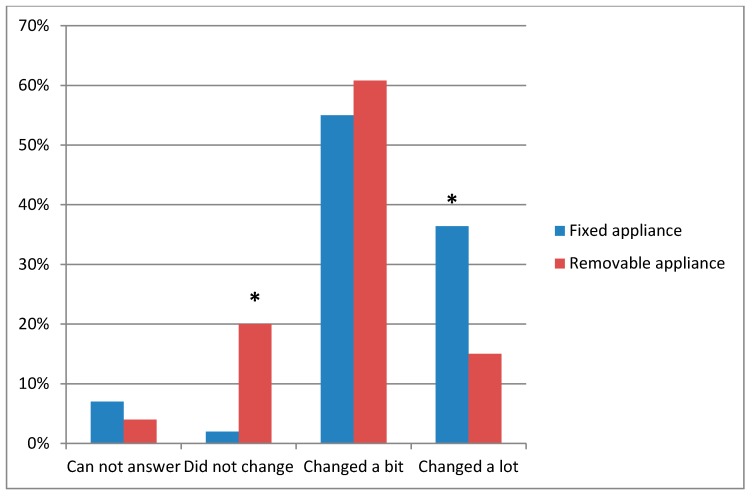
Changes in oral hygiene habits among patients receiving orthodontic treatment. Chi-square test, comparing results by different type of receiving orthodontic treatment (fixed and removable appliances); * —statistically significant differences (*p* = 0.002).

Considering the changes in oral hygiene habits among patients receiving orthodontic treatment, statistically significantly more patients receiving orthodontic treatment with removable appliances than patients receiving orthodontic treatment with fixed appliances reported that their oral hygiene habits did not change (20.0% vs. 2.3%), whereas significantly more patients receiving orthodontic treatment with fixed appliances answered that their oral hygiene habits changed a lot than patients receiving orthodontic treatment with removable appliances (36.4% vs. 15.0%), respectively (*p* = 0.002, Figure 1).

## 4. Discussion

Patients receiving orthodontic treatment reported using more self–care adjunctive measures, such as auxiliary interproximal measures, tongue brushing and rinsing the mouth after meals, compared to non-orthodontic patients in this study. In addition, significantly more orthodontic patients had professional oral hygiene than non-orthodontic patients.

Considering the previous studies carried out in Lithuania, the prevalence of malocclusion was high (84.6%) and the need for orthodontic treatment varied from 33.4% to 42.6% among Lithuanian adolescents [28,29,30]. Moreover, 47.5% of 11–15-year-old schoolchildren in Lithuania reported having malposed teeth [31]. Currently, there are limited data about oral hygiene habits among patients receiving orthodontic treatment in Lithuania. Moreover, adolescents tend to maintain a lower quality of tooth brushing and to have more dental plaque than adults [32]. Thus, it is essential to assess oral hygiene habits among adolescents receiving orthodontic treatment.

Quality of oral hygiene is related to several factors such as frequency and duration of tooth brushing, type of toothbrush and toothpaste used, and selected auxiliary oral health measures. In this study, a high prevalence of participants followed the recommendations for tooth brushing frequency and duration. Thus, two-thirds of patients receiving orthodontic treatment brushed their teeth at least twice a day and for at least 2 min. Meanwhile, other studies showed a lower share of participants following the tooth brushing recommendations, and tooth brushing once a day was found to be prevalent among participants in Saudi Arabia [33] and India [34].

In this study, a manual toothbrush was the first choice for almost all participants, and only 4.5% of subjects reported using a powered toothbrush. Furthermore, other studies were in line with our findings [35,36]. The literature reveals controversial results about the effectiveness of different types of toothbrushes for dental plaque removal. Sharma et al. [37] found that all the selected toothbrush types (manual orthodontic, powered and sonic) were equally effective in the control of biofilm for patients with fixed orthodontic appliances, while Mazzoleni et al. [38] found a higher effectiveness of the electric toothbrush over the manual toothbrush among patients with rapid palatal expanders, especially in the early months of use. In addition, another study showed that the oscillating rotating electric toothbrush, especially with an orthodontic brush head, demonstrated a significantly higher effectiveness for plaque removal over the manual brush, among patients with fixed orthodontic appliances [39]. Although some authors claim that powered toothbrushes may maintain better gingival health status than manual toothbrushes in patients receiving orthodontic treatment, the results of several performed meta-analyses revealed that the relevant scientific evidence was inconclusive due to insufficient follow-up periods and that the effectiveness of powered toothbrushes was not investigated in an evidence-based manner [40,41]. Considering the “novelty effect”, participants are motivated to use an electric toothbrush for the first time in a short-term period, but later this personal interest in novelty fades [38]; thus, the type of selected toothbrush does not play an essential role in plaque removal.

In this study, patients did not pay attention to the kind of toothpaste used, because nearly half of the participants did not know which type of toothpaste they used, and only 37.2% of subjects reported using toothpaste with fluoride. Fluoride toothpaste is recommended to orthodontic patients due to its potential to decrease the risk of the development of caries, especially when a different fluoride regime of toothpastes (1450 ppm and 5000 ppm) is combined [42]. Findings from other studies revealed a higher usage of toothpaste with fluoride, varying from 61% to 81.9% [43,44]. The low attention paid to toothpaste selection might be price-driven or due to the peculiarities of young patient age and a low awareness of patients in our study.

A regular usage of interproximal measures leads to higher action and maintenance of self-efficacy, a lower level of biofilm and better periodontal tissue status in orthodontic patients [45,46]. However, several studies revealed that the majority of patients were not familiar with a regular usage of interdental aids, such as dental floss or an interdental brush [34,35,36,43,46,47,48]. This study suggested a reason for optimism, since 56% of participants receiving orthodontic treatment reported using interproximal measures, while significantly less non-orthodontic patients reported the same oral behavior. This might be explained by the regular instruction about oral hygiene provided by an oral hygienist or orthodontist during follow-up visits.

Mouth rinsing is recommended as an addition to maintain good oral hygiene, and it might improve gingival health for patients receiving orthodontic treatment [49]. In this study, most of the orthodontic patients with fixed appliances reported mouth rinsing after meals, while this habit was less prevalent among the control group participants. The findings of other studies revealed that less than half of patients used mouthwash [34,35,36,43,44].

Various studies suggest that females have better attitudes and habits toward oral hygiene than males due to a greater interest in their appearance [50]. Males tend to have significantly more dental plaque than females [32]. In addition, another study showed that a significantly higher gingival bleeding was observed among males [46]. Similarly, in this study more girls used auxiliary interproximal measures than boys, and the findings were in line with a study carried out in Jordan [51]. Consequently, low scores of plaque indices were related to a frequent use of a proxy brush, higher intention toward the usage of the proxy brush, female gender and older age [45].

A structured follow-up during orthodontic treatment improves the maintenance of optimal oral hygiene [52]. Similarly, a good oral hygiene status is defined as a predictor of adherence to orthodontic treatment [53]. In this study, significantly more patients receiving orthodontic treatment (45.3%) reported attending oral hygienists than participants in the control group (31.5%, *p* = 0.024), while a study carried in Poland showed the same trend [36]. A better patient attitude toward oral hygiene might be related to the type of orthodontic treatment.

This study revealed that the oral hygiene habits of patients were changed according to the type of orthodontic treatment. Therefore, statistically significantly more patients receiving treatment with fixed appliances reported that they changed their oral hygiene habits a lot than patients with removable appliances. In addition, another study carried out in Lithuania showed that age played a role in oral hygiene changes, and patients receiving orthodontic treatment with fixed appliances aged 16 to 18 changed their habits more intensively than participants aged 12 to 15 [54].

### Strengths and Limitations

This study enrolled patients aged 10–17 years who attended the Department of Orthodontics, LSMU Hospital, with a response rate of 97%, which can be considered high. An estimation of the sample size was made based on the number of registered appointments for consultation or orthodontic treatment in the Department of Orthodontics, LSMU Hospital. Moreover, the control group was selected from patients attending the clinic for consultation and not receiving orthodontic treatment. The homogeneity of the population strengthens the comparability between groups but reduces the external validity and generalizability. The limitations of this study should be considered. This study is not representative. The data were collected with a self-reported questionnaire, while the quality of oral hygiene and presence of dental plaque among participants were not assessed by investigators, and the possibility of both intentional and unintentional misreporting can compromise the validity and reliability of the findings. Finally, patients receiving orthodontic treatment are encouraged to oral hygiene instructions and monitored regularly by their orthodontist, thus the quality of oral hygiene should be improved over the whole period of orthodontic treatment.

## 5. Conclusions

In this study, patients receiving orthodontic treatment used self-care adjunctive aids, such as auxiliary interproximal measures, tongue cleaning, and mouth rinsing after meals, more scrupulously than patients not receiving orthodontic treatment. Considering changes in oral hygiene habits, more patients with fixed orthodontic appliances reported significantly changed oral hygiene habits than patients with removable orthodontic appliances. Meanwhile, more patients receiving orthodontic treatment with removable appliances reported that they had not changed their oral hygiene habits than patients receiving orthodontic treatment with fixed appliances.

## Figures and Tables

**Table 1:** Demographic characteristics of participants.

	Control Group N (%)	Patients Receiving Orthodontic Treatment N (%)	Total N (%)
**Gender (Missing N = 1)**			
Girls	65 (53.7)	99 (58.6)	164 (56.6)
Boys	56 (46.3)	70 (41.4)	126 (43.4)
Total N (%)	121 (100)	169 (100)	290 (100)
**Age (Years)**			
10–12	65 (53.7)	80 (47.1)	145 (49.8)
13–17	56 (46.3)	90 (52.9)	146 (50.2)
Total N (%)	121 (100)	170 (100)	291 (100)
**Place of Residence (Missing N = 4)**			
Urban	88 (73.9)	125 (74.4)	213 (74.2)
Rural	31 (26.1)	43 (25.6)	74 (25.8)
Total N (%)	119 (100)	168 (100)	287 (100)

*p* > 0.05, Chi-square test.

**Table 2:** Oral hygiene habits among participants visiting the Department of Orthodontics at Lithuanian University of Medical Sciences (LSMU) Clinics.

	Control Group N (%)	Patients Receiving Orthodontic Treatment N (%)		Total N (%)	*p*-Value
		Fixed Appliance	Removable Appliance		
**Frequency of Tooth Brushing (missing N = 4)**					
Irregularly	16 (13.2)	1 (2.3)	11 (9.0)	28 (9.8)	0.171
Once a day	36 (29.8)	10 (22.7)	36 (29.5)	82 (28.6)	
≥2 times a day	69 (57.0)	33 (75.0)	75 (61.5)	177 (61.6)	
Total N (%)	121 (100)	44 (100)	122 (100)	287 (100)	
**Duration of Tooth Brushing (missing N = 5)**					
≤1 min	18 (15.0)	8 (18.2)	29 (23.8)	55 (19.2)	0.220
≥2 min	102 (85.0)	36 (81.8)	93 (76.2)	231 (80.8)	
Total N (%)	120 (100)	44 (100)	122 (100)	286 (100)	
**Type of Toothbrush (missing N = 4)**					
Manual	115 (95.0)	42 (95.5)	117 (95.9)	274 (95.5)	0.899
Powered	6 (5.0)	2 (4.5)	5 (4.1)	13 (4.5)	
Total N (%)	121 (100)	44 (100)	122 (100)	287 (100)	
**Type of Toothpaste (missing N = 4)**					
With fluoride	44 (36.1)	20 (45.5)	43 (35.3)	107 (37.2)	0.926
Fluoride-free	16 (13.1)	6 (13.6)	21 (17.2)	43 (14.9)	
Do not know	62 (50.8)	18 (40.9)	58 (47.5)	138 (47.9)	
Total N (%)	122 (100)	44 (100)	122 (100)	288 (100)	
**Mouth Rinsing After Meals (missing N = 11)**					
Yes	62 (54.4)	35 (79.5)	77 (63.1)	174 (62.1)	0.013
No	52 (45.6)	9 (20.5)	45 (36.9)	106 (37.9)	
Total N (%)	114 (100)	44 (100)	122 (100)	280 (100)	

Chi-square test, comparing results between the control group and patients receiving orthodontic treatment (fixed appliances and removable appliances).

**Table 3:** Usage of auxiliary self-care measures and attendance to dentists and oral hygienists among participants visiting the Department of Orthodontics at LSMU Clinics.

	Gender * N (%)		Area ** N (%)		Orthodontic Treatment *** N (%)		*p*-Value
	Boys	Girls	Rural	Urban	Yes	No	
**Usage of Auxiliary Interproximal Measures**							
Yes	53 (42.7)	89 (54.6)	32 (44.4)	108 (50.9)	93 (56.0)	49 (40.8)	*p* = 0.133 * p = 0.017 ** p = 0.015 ***
No	71 (57.3)	74 (45.4)	40 (55.6)	104 (49.1)	73 (44.0)	71 (59.2)	
Total N (%)	124 (100)	163 (100)	72 (100)	212 (100)	166 (100)	120 (100)	
**Tongue Cleaning**							
Yes	42 (33.3)	81 (49.4)	29 (39.2)	91 (42.7)	81 (47.6)	42 (34.7)	*p* = 0.006 * p = 0.595 ** p = 0.028 ***
No	84 (66.7)	83 (50.6)	45 (60.8)	122 (57.3)	89 (52.4)	79 (65.3)	
Total N (%)	126 (100)	164 (100)	74 (100)	213 (100)	170 (100)	121 (100)	
**Dental Appointments**							
Irregular	15 (12.3)	35 (22.3)	15 (21.7)	33 (15.9)	27 (16.3)	23 (20.2)	*p* = 0.05 * p = 0.035 ** p = 0.420 ***
Twice a year	41 (33.6)	38 (24.2)	26 (37.7)	53 (25.6)	44 (26.5)	35 (30.7)	
Once a year	66 (54.1)	84 (53.5)	28 (40.6)	121 (58.5)	95 (57.2)	56 (49.1)	
Total N (%)	122 (100)	157 (100)	69 (100)	207 (100)	166 (100)	114 (100)	
**Professional Oral Hygiene**							
Yes	44 (37.3)	62 (41.9)	24 (36.9)	82 (41.4)	72 (45.3)	34 (31.5)	*p* = 0.446 * p = 0.522 ** p = 0.024 ***
No	74 (62.7)	86 (58.1)	41 (63.1)	116 (58.6)	87 (54.7)	74 (68.5)	
Total N (%)	118 (100)	148 (100)	65 (100)	198 (100)	159 (100)	108 (100)	

* comparison between genders, Chi-square test; ** comparison between places of residence, Chi-square test; *** comparison between patients receiving orthodontic treatment and the control group, Chi-square test.

**Table 4:** Factors explaining the oral behavior of patients receiving orthodontic treatment, as assessed by means of univariate logistic regression of the participants (N = 291) visiting the Department of Orthodontics at the LSMU Hospital.

	OR	95% CI	*p*-Value
Tongue Brushing			
Yes	1.712	1.059–2.767	0.028
No	1	-	-
Auxiliary Measures			
Yes	1.797	1.118–2.887	0.015
No	1	-	-
Mouth Rinsing After Meals			
Yes	1.707	1.048–2.781	0.032
No	1	-	-

OR—odds ratio; CI—confidence interval.

## Abbreviations

LSMU	Lithuanian University of Medical Sciences
M	Mean Score
SD	Standard Deviation
OR	Odds Ratio
